# The moral and social narratives of sexual and reproductive health in Kenya: a case of adolescents and young people pre- and within the MDG era

**DOI:** 10.1186/s12978-020-00930-x

**Published:** 2020-05-26

**Authors:** Elsie Akwara, Priscilla Idele

**Affiliations:** 1 World Health Organization, Department of Sexual and Reproductive Health and Research/Human Reproduction Programme, Geneva, Switzerland; 2 United Nations Children’s Fund Office of Research, Florence, Italy

**Keywords:** Reproductive health, Adolescents, Sexual health, MDGs, Kenya, HIV/AIDS

## Abstract

**Background:**

The role of global initiatives in catalyzing change within national contexts is complex and less understood. Addressing adolescent sexual and reproductive health in Kenya requires concerted efforts of both state and non-state actors and more importantly, a supportive environment. This paper deconstructs the moral and social narratives of adolescents’ and young people’s sexual and reproductive health (AYSRH) in Kenya as driven by the powerful discourse and ideologies pre- and within the Millennium Development Goal (MDG) era.

**Methods:**

Literature was systematically searched in PubMed and Medline with policy documents obtained from government agencies from the pre-MDG period (2000 and earlier) and within the MDG period (2001–2015). Literature with a substantial focus on SRH were eligible if they captured the different facets of ASRH in Kenya and sub-Saharan Africa (SSA). The results were reviewed and synthesized to disentangle the moral and social narratives of AYSRH in Kenya with an MDG lens.

**Results:**

The evolution of AYSRH policies and programmes in Kenya was gradual and largely shaped by prevailing development threats and moral and social narratives. Pre-MDG period was dominated by issue-based policies of population growth and high fertility rates, with a focus on married population with strong cultural and religious barriers to AYSRH; early to mid-MDG was mainly influenced by the threat of HIV/AIDS, culminating in the first Adolescent Reproductive Health and Development Policy in 2003. However, the policies and subsequent programmes focused on abstinence only and medical narratives, with persistent religious and cultural opposition to AYSRH. Late-MDG saw more progressive policies (these are policies that refer to those that tends towards acceptance of liberal social reforms and which sometimes are contrary to entrenched social norms, beliefs and practices), high government commitment and a refocus on SRH issues due to sustained early childbearing, culminating in the revised Adolescent Sexual and Reproductive Health Policy of 2015.

**Conclusion:**

Debates in the translation of global goals and commitments to policy and practice at country level need to account for national level realities in AYSRH reforms. The findings contribute to critical evidence for strategic policy and programming approaches for AYSRH in Kenya and in SSA and for the realization of their rights within the context of sustainable development goals.

## Plain English summary

Global goals and initiatives are presumed to provide a framework for development partners and governments for accelerating progress towards eliminating poverty and ensuring equitable and sustainable development. However, the level of political priority at the national level varies given domestic competing interest within the context of adolescents’ and young people’s sexual and reproductive health (AYSRH) issues. This paper deconstructs the moral and social narratives of adolescents’ and young people’s sexual and reproductive health in Kenya as driven by the powerful discourse and ideologies pre- and within the Millennium Development Goals (MDG) period. Findings reveal that there has been progressive evolution of AYSRH policies pre and within the MDG era, where the pre- MDG was mainly concerned with population growth, whereas the early to Mid- MDG period was dominated by HIV-specific policies and strategies. Although the MDGs provided a political impetus for AYSRH reform policies, the dominant narrative constraining AYSRH reforms were entrenched in the strong moral, cultural and religious discourse in the country. The late-MDG period saw more progressive policies, high government commitment and a refocus on SRH issues, culminating in the revised Adolescent Sexual and Reproductive Health Policy of 2015. The findings reveal that debates in the translation of global goals and commitments to policy and practice at country level need to account for national level realities in AYSRH reforms. The findings contribute to critical evidence for strategic policy and programming approaches for AYSRH in Kenya and in sub-Saharan Africa.

## Background

Global and regional initiatives are assumed to provide a framework for development partners and governments for accelerating progress towards eliminating poverty and ensuring equitable and sustainable development; However, health initiatives vary in the amount of political priority they receive and the degree to which policies and strategies are formulated to address pressing issues at the national level [1]. Kenya is signatory to these momentous goals, among other similar commitments and has aligned the overall national development agenda, as well as the SRH policies and programmes with the global and regional commitments. This includes alignment with emerging SRH rights and the transformational changes in the Kenyan national health system stemming from the devolved government and over the years, has re-shaped and revised various instruments to address the AYSRH needs. However, progress towards the goals at country level is beyond ratification; it requires concerted efforts of both state and non-state actors and more importantly, a supportive environment.

Although there have been some notable improvements in AYSRH in Kenya, there still remain large gaps in terms of access and use of services. Adolescent girls and young women lag behind in most SRH indicators and there is limited understanding of what influenced the AYSRH environment pre- and within the MDG period. There is no specific documentation and systematic assessment of the key factors, especially social and religious ideologies that shaped the SRH of adolescents and young people in Kenya from the lens of the MDGs. While political willingness for advancing the AYSRH existed from 1999 when HIV/AIDS was declared a national disaster by the President at the time, resistance from religious and cultural groups persisted and did not allow for sex education or use of contraception among other issues related to SRH for young people, especially adolescents. The adoption of the SDGs and other additional initiatives such as FP2020 (2012), Girls Not Brides (2011), Together for Girls (2009), and Global Strategy on Women, Children and Adolescent Health (2016–2030) among others, call for a better understanding of what influenced the SRH of adolescents and young people pre- and within the MDG period to better inform the translation of the SDGs into national policy and practice in Kenya. The SDGs offer Kenya an unprecedented opportunity to build on the achievements of the MDGs and to course correct on any shortcomings from past experiences.

This paper therefore deconstructs the moral and social narratives of AYSRH in Kenya in order to understand norms, ideology and discursive power influencing AYSRH policies and programmes in Kenya. More specifically, it seeks to answer the following question: *How did power relations and context enable or influence the realization of AYSRH pre and within the MDG era in Kenya?*

The paper is based on the premise that political and socio-cultural norms and ideologies shaped the SRH policies and programmes for adolescents and young people in Kenya pre- and within the MDG era. The assessment is not aimed at a causal investigation nor assessment of the impact of the MDGs, but to understand how and what shaped the evolution of the AYSRH environment in relation to global and regional goals and commitments versus Kenya’s national needs and priorities for adolescents and young people pre-MDG and within the MDG period.

## Methods

Data presented in this paper were collected through a review of academic and grey literature to contextualize the AYSRH environment in Kenya. The literature search was conducted using Google Scholar, PubMed, Cochrane Database and MEDLINE with the following key words and phrases: sexual and reproductive health, sexual coercion, HIV, contraceptive use, family planning, youth friendly services, SRH policies and AND adolescents, youth, teens, young people IN combination with Kenya, sub- Saharan Africa, East Africa, culture, family dynamics, and ethnicity. A review of published global and regional goals and national policies and programme documents and other organisational and institutional publications was also conducted to contextualize the AYSRH environment in Kenya.

AYSRH policy and programme documents were obtained online from government agencies (Department of Reproductive Health [DRH], National Council for Population Development [NCPD], National Aids Control Council [NACC], National Aids and STI Control Program [NASCOP], the Kenya Law Reform commission and the National Council for Law Reporting. Documents related to regional and international agreements, conventions or declarations were searched and accessed from websites of key organisations such as UN agencies and the African Union.

Information gathered and assessed was from the pre-MDG period (2000 and earlier) in order to contextualize AYSRH and situate within the MDG period (2001–2015). A total of 35 documents were reviewed- 22 from peer reviewed journals and 13 from grey literature; a total of 12 global initiatives/strategies/policies were included with 39 policies and programmes at the national level.

Policies, programmes and strategies related to sexual violence, sexual and life skills education in school, gender equality, access to and use of SRH services, HIV and AIDS, youth friendly services (YFS), the national constitution, youth policy, health policy and AYSRH were assessed on how they have evolved over time.

### Data analysis

The analysis drew heavily from the available literature to disentangle the moral and social narratives of AYSRH in Kenya with an MDG lens. Data and other information on AYSRH macro environment were extracted from the peer-reviewed articles and grey literature. To align with the research aim, information was organized according to three MDG phases: (i) Phase I: Pre-MDG period (2000 and earlier) (ii) Phase II: Early to Mid- MDG period (2001–2009) (iii) Phase III: Late- MDG period (2010–2015). The pre-MDG era provides an understanding of how the issue of AYSRH has evolved overtime and the challenges in the processes leading to the development of the first AYSRH policy in 2003 and the subsequent revision in 2015. The phased approach allows capture of the nuances between the different MDG.

## Results

### How did power relations and context enable or influence the realization of AYSRH pre and within the MDG era in Kenya?

The evolution of AYSRH policies and programmes in Kenya was gradual and was largely shaped both by prevailing national development threats and priorities, as well as, the political, social, cultural and religious ideologies [See Table 1]. The subsequent sections present the results of the assessment in line with the research question in three MDG phases.

**Table 1:** Summary of main development issues, national policies and strategies and barriers pre-MDG and within the MDG period

Period	Main Development issues	Kenya National Policies/Strategies	Main barriers/enablers to AYSRH
Pre-MDG (Earlier to 2000)	High population growth; High fertility; Family planning for married couples	Family Planning Programme, 1967National Population and Development Policy, 1984National Guidelines for Family Planning, 1984National AIDS and STIs Control Programme, 1987Center for the Study of Adolescents, 1988Return to school Policy, 1994Kenya Health Policy Framework (KHPF 1994–2010)National Reproductive Health Strategy (1997–2010)National Population policy for Sustainable Development, 1999National AIDS Control Council established, 1999Kenya National Population Policy for Sustainable Development, Sessional Paper No.1, 2000	Strong traditional political influence and policies; Male hegemony; Sociocultural norms and beliefs; Religious ideology
Early to Mid-MDG (2001–2009)	HIV/AIDS	The National HIV/AIDS Strategic plan, 2000–2005 Condom policy strategy, 2001–2005 Kenya Children’s Act, 2001Adolescent Reproductive Health & Development Policy, 2003Education re-entry policy, 2003The Persons With Disabilities Act, 2003Policy on HIV in the Education Sector, 2004National Guidelines for Provision of Youth-Friendly Services, 2005Kenya HIV & AIDS Prevention and Control Act, 2006 Sexual Offences Act, 2006National Youth Policy, 2006National Reproductive Health Policy: Enhancing Reproductive Health Status for All Kenyans, 2007Gender Policy in Education, 2008Kenya Vision 2030, most notably the First Medium Term Plan, 2008–2012National HIV/AIDS Policy, 2009National Reproductive Health and HIV/AIDS integration strategy, 2009National Condom Policy and Strategy, 2009–2014	Strong political influence; Male hegemony; Sociocultural norms and beliefs; Religious ideology; Medical narrative; Evolution of civil society advocacy and influence
Late MDG (2010–2015)	Sustained high teenage pregnancy; Early marriage; Youthful population	The Constitution of Kenya (revised), 2010Reproductive Health Communication Strategy, 2010–2012 Kenya National School Health Strategy, 2010–2015 Prohibition of Female Genital Mutilation Act, 2011 Gender Policy, 2011National Communication Strategy for Community Health Services, 2012–2017 Sessional Paper No.3 on Population Policy for National Development, 2012Population Policy for National Development, 2012–2030 Education Sector Policy on HIV and AIDS, 2013- revised to align with the revised constitution and vision 2030National Gender-Based Violence, 2014 The Marriage Act, 2014Revised Adolescent Sexual and Reproductive Health Policy, 2015	Strong political will and commitment; Progressive policies; Resource allocation, SRH rights narrative; civil society voice and influence; Male hegemony; Sociocultural norms and beliefs; Religious ideology

#### Phase I: pre-MDG period (2000 and earlier)

The pre-MDG period was mainly characterized by issue based SRH policies and programmes that focused on reducing high population growth and fertility, and the promotion of family planning (FP) among married individuals as evidenced in the first national FP programme [2]. Although advocacy efforts to address SRH issues of adolescents and young people was prominent through the work of civil society groups in the 1980s and 1990s, the absence of explicit SRH health policy documents, programmes or strategies, clinical based FP programme and the prioritisation of economic development in the country, meant that the SRH needs of adolescents and young people, particularly the unmarried would remain neglected. The first population policy in the country was initiated as a result of the international population movement and donor pressure with limited government interest; the lacklustre effort and poor ownership of the government in tackling general population issues meant that AYSRH issues were far from being included in the agenda of the country’s development focus [2, 3].

Strong opposition to population control (including use of contraceptives) was apparent in the policy environment in the 1960s and 1970s and the prevailing cultural and religious attitudes (pro-natalist and anti- Comprehensive Sexual Education (CSE) sentiments on the cultural front on the premise that the latter perpetually leads to early sexual debut and anti-Adolescent and youth friendly services (AYFS) on the religious front due to concerns of pre-marital sexual activities) sustained and supported high fertility. Any attempt to address SRH issues for adolescents and young people emphasized abstinence only [4]. The strong cultural and religious norms and values in this period worked against adolescent SRH debates in the country. Only one independent non-governmental organization (NGO) – the Centre for the Study of Adolescents (CSA) was established to specifically advocate for adolescents’ SRH issues, including HIV/AIDS in the late 80s, and was also instrumental in the reform efforts leading to Kenya’s first Adolescent Reproductive Health and Development (ARH&D) Policy in 2003. Before the 1980s, there was no evidence of attempts in the previous two decades of Kenya’s pro-natalist effort to design policy interventions that addressed AYSRH issues.

Although a change in government administration in 1982 saw the government take charge in addressing high population growth rate, seen as a serious impediment to economic and social development, AYSRH issues continued to be neglected as they continued to register a high fertility rate [5]. The focus of the programme and the population policy were mainly for reducing population growth and targeted married individuals with contraceptive services and information, education and communication (IEC) provided mainly by the private sector - systematically excluding the younger population who were less likely to afford the costs at the private sector. Despite research by CSA on adolescents revealing high levels of teenage pregnancy and unsafe abortion (about 252,000 abortions among 15–19 year olds) throughout the 1990s, the powerful political opposition, underpinned by moral and cultural interests persisted [6, 7].

International efforts to address SRH issues in many developing countries, including in Kenya was prominent in the 1994 International Conference on Population and Development (ICPD), which reframed SRH as a human rights issue [8]. Other global efforts to address ASRH issues pre-MDG included the child rights framework- article 20 of the Convention on the Rights of the Child (CRC) [9], the 1995 Beijing Platform for Action [10] and the Convention on the Elimination of All Forms of Discrimination against Women (CEDAW) [11]. This lent legitimacy to local advocacy efforts in Kenya to address adolescents’ and young people’s SRH needs. It also paved the way for NGOs to embark on interventions and programmes targeting adolescents and young people. While Kenya adopted the 1994 ICPD, the Minister for Planning in Kenya at the time indicated that it was not possible to embrace AYSRH due to the prevailing religious and cultural values in the country [12]. In the same year of the ICPD, Kenya passed a school re-entry policy for young mothers. The inception of the school re-entry policy efforts can be traced back to the International protocols that established Education for All (EFA) agenda in Thailand (1990), the National Conference on Girls held in Nyeri (1992) and the subsequent Machakos National Symposium (1994) that saw the setting up of the gender and education task force and the Girl Child project [13].

The largest program implemented in this period (The Kenya Adolescent Reproductive Health programme [KARHP]) focused on adolescents specifically (10–19 years). It was the first initiative that involved the government Ministries, resulting in a decrease in the proportion initiating sexual intercourse, an increase in knowledge of sexually transmitted infections, particularly HIV/AIDS awareness which was near universal [14]. However, the low use of condoms and increase in the proportion of pregnant girls at the time suggests that despite a somewhat conducive climate to operationalise the ICPD in the country, other factors working at the macro level were inhibiting full access to SRH services and information to adolescents and young people.

Furthermore, the policy for re-entry to formal schooling by young mothers was more of a reactionary and passive step, bowing to the influence of the international community [15]. Thus, the moral narratives in the country that viewed teenage motherhood as disgraceful continued to hinder the implementation and dissemination of the policy in the country. In fact, 77% of the schools were against the re-entry policy and the negative perceptions from the community, stigma and lack of awareness of the policy among stakeholders exacerbated the issue of young mothers returning to formal schooling [16–18]. In some cases, marriage was sought either voluntarily or coerced by parents in order to legitimize pregnancies and childbirth. The policy lacked guidelines and schools were afraid of being ostracised if they allowed re-entry of young mothers [13, 19].

An attempt at developing an adolescent RH policy was done in 1999 but was rejected as evidenced by the non-progressive male policy makers who dominated the government administration at the time [7]. Although the SRH rights narrative was highlighted in the ICPD, adolescent’s right to SRH information and services were not emphasised in advocacy efforts for fear of opposition by the religious network in the country. Another attempt to address AYSRH issues was made in 1997 by the Civil Society Organisations (CSOs), who sought to introduce a bill on Family Life Education that would have paved the way for the provision of sexuality education in school but was blocked by the Kenyan administration [7]. The attempt of scaling up the family life education countrywide saw the peak of the controversy particularly among religious groups, whereby books used in the pilot programme were burnt in the streets, as well as, condoms (ibid). AYSRH issues were strongly opposed by top government and political leadership as seen by views expressed by the president at that time on the immorality of sexuality education programme for children [20]. The books used in the family life education were condemned as being immoral by religious groups [21].

This period also saw an attempt to address sexual violence in the country, whereby news of 70 girls being raped, leaving 19 dead in a secondary school pushed CSOs to advocate for policy reforms, but remained a highly contested issue that did not see a policy realised until in the early MDG period [22]. Efforts to introduce similar policies that would address AYSRH such as banning of Female Genital Mutilation (FGM) in 1996 were not successful due to strong cultural narratives that deemed banning FGM as deviating from the Kenyan culture [23]. The CSA was dominated by medical professionals, thus there was a prevailing medical narrative, in which medical practitioners advocated for access to AYSRH services for improving health outcomes among adolescents and young people, rather than as a fundamental human rights issue. The voices of other key stakeholders such as human rights advocates that could have been champions of SRH as a human right were absent. Thus the medical narrative rested on adolescent’s lack of SRH information and services resulting to poor AYSRH outcomes., Despite the promulgation of several SRH policies in the pre-MDG period, adolescents and young people were continuously left out of the country’s SRH policies and strategies. Kenya’s male-dominated Senate and National Assembly marginalised the issues of women and girls [7].

#### Phase II: early to mid- MDG period (2000–2009)

The advent of HIV/AIDS as a national emergency in the early 2000s created partial policy space to address adolescents’ SRH issues as religious and cultural opposition became less vocal, as the epidemic ravaged the population. This seemed to be a blessing in disguise as it led to positioning of the issues of adolescents’ SRH on top of the national agenda; hence diverting the attention from the development focused MDGs to the prevailing national threat – HIV/AIDS [2, 3].

Although reform efforts leading to Kenya’s first adolescent reproductive health (ARH&D) policy of 2003 were initiated in the early 1990s, advocacy efforts to cascade support excluded issues perceived as more sensitive, such as provision of contraceptives and abortion services to adolescents. Instead, the focus was on the need of service provision related to HIV/AIDS and general SRH information to reduce opposition from the religious and political fronts [7]. This reflected the power of some of the stakeholders such as the Christian Health Association of Kenya (CHAK) which was against abortion entirely and that also dominated the development process of the first ARH&D policy. Politicians were also influential in the development policy and excluded the voices of adolescents and grassroots organisations. The human rights approach to SRH, which was dominant at the international level, was greatly contested and marginalised at the national level in Kenya, especially as it pertained to AYSRH, making it easy to marginalise the human rights framework and approach to programming [7, 24]. As a result, the first adolescent RH policy that was passed in 2003 was watered-down with broad statements that did not clearly address the provision of SRH services and information to adolescents and young people. There was prioritisation of HIV/AIDS education, but no mention of contraceptives or comprehensive sexuality life skills. After the passing of the policy, the plan of action (POA) guidelines for the policy delayed by 2 years, showing poor prioritization on AYSRH issues. The ARH&D policy faced various implementation challenges, including delayed development of the Plan of Action, inadequate dissemination, poor leadership and coordination and low stakeholder and youth involvement, inadequate resources, lack of political will and cultural and religious barriers to issues related to AYSRH [24].

The ARH&D policy recognised the rights of the youth as in various AYSRH policies during the early- to the mid-MDG period, however, it was still a highly contested issue in the country among the religious networks that saw provision of SRH information and services to adolescents as immoral [25]. Although there was a renewal of efforts to address AYSRH in the early-MDG period as opposed to the pre-MDG period, some groups were still excluded in the policy development process, such as, girls and human rights groups, and dominated by the medical practitioners.

The launch of the MDGs also saw the first policy that specifically addressed children’s needs in the country through the Kenya Children’s Act of 2001. Although the Act was not necessarily enacted because of the MDGs solely, it was mainly influenced by the global and regional commitments. This was the first Children’s Act in the country to specifically give children a right to health and protection from sexual exploitation and abuse. Even though the MDG goals arose in 2000s, it was not until 2004 when the government agreed to implement “Mainstreaming MDGs in Kenya’s Development Process” or the MDG project [26]. The framework was rather an external requirement guiding access to international aid rather than the competing core development priorities in the country. The subsequent policies that ensued (e.g. the first adolescent SRH policy) point to the direction the MDG framework emphasized which was on development, relegating the issues of rights in AYSRH at the bottom. However, the emphasis on some health indicators (MDG 5 & 6) brought to the forefront issues of SRH that touched on adolescents and young people’s health.

The development of the first ARH&D policy and various subsequent policies that addressed AYSRH were also catalysed by the 2002 change of government administration that saw an opening window for other SRH policy reforms. The change in administration saw the coming into parliament of some progressive Members of Parliament (MPs), who managed to pass the ARH&D policy in 2003 in order to respond to the concerns raised about mainstreaming adolescent health and development issues. The new government administration also brought an impetus of change that helped mobilize action to address sexual offences culminating in the Sexual Offences bill which began in the pre-MDG period and only passed in 2006 [2]. However, given the unsupportive environment in the mid-2000s, advocacy efforts achieved only an amendment to the Penal Code pertaining to minors that protected the minor’s identity [25]. The Sexual Offences Bill sought to criminalise all sorts of sexual violence, including rape in marriage and unwelcome sexual advances, which contravened with the dominant cultural narrative in which the bill was seen by many male Members of Parliament (MP) that dominated the parliament as deviating from the African culture [22].

The change in government administration in 2002, as well as, the adoption of the MDGs also saw the establishment of the *Families Matter!* nationwide programme in 2003 (funded by USAID through The President’s Emergency Plan for AIDS Relief (PEPFAR)) with an aim of improving parent-child communication about sexual risk reduction. The acceptance of this programme in rural areas of the country demonstrated that the deeply held cultural narratives at the household and community level that worked to block AYSRH initiatives previously could be countered through tailored interventions that not only targeted adolescents and young people in isolation, but included their custodians and gatekeepers. However, the reliance on donor support meant that the country had to prioritise donor’s interests at the expense of other AYSRH issues, which played a role in the moralised development process concerning adolescents’ SRH in the policy making. The US government’s global gag rule meant that there was no support on abortion-related work and supported abstinence only programmes for unmarried adolescents and youth [27].

Despite barriers at the cultural, social and political fronts, the early- to mid-MDG period saw more forward-looking policies that included adolescents and young people beyond the MDGs, such as Vision 2030 which contained the main economic (incorporating MDGs 1,3 and 8), social (incorporating MDGs 2 and 4–7) and political (incorporating the United Nations Millennium Declaration V that entails human rights, democracy and good governance) goals of the country. The Vision was an implementation of the MDGs into a longer-term national policy framework, projecting 15 years beyond the MDGs. The 2030 vision for gender, youth and vulnerable groups is anchored on equity in power and resource distribution between the sexes, improved livelihoods for all vulnerable groups, and responsible, globally competitive and prosperous youth. But there is nothing specifically on AYSRH. However, the vision aided, in part, the process that led to a revised adolescents and reproductive health policy in 2015 that mainstreamed AYSRH issues into the national development agenda.

#### Phase III: late MDG period (2010–2015)

The late MDG period was dominated by more progressive and inclusive policies and programmes. The stall in fertility and child mortality outcomes and sustained early childbearing was an awakening for the government to refocus on SRH issues, with more attention to the SRH needs of adolescents and young people. More attention was focused on addressing problems pertaining to the youth as evidenced by development of policies such as gender policy (2011), education sector policy on HIV and AIDS (2013), gender-based violence (2014), the Marriage Act (2014) and the Reproductive Health Care Bill (2014) (waived parental consent to provision of RH services to adolescents). These policies used the global goals of the MDGs to provide a political impetus and framework for galvanizing the community at the national level to work on adolescents’ and young people’s issues. The country’s Vision 2030 and the revised 2010 Constitution catalysed inception of these policies, focusing mainly on mainstreaming AYSRH into the country’s development agenda given the gender inequality in the country as well as the disproportionate number of adolescents and young women affected by HIV and AIDS. All of these policy instruments promoted AYSRH and rights such as ending early marriage (age of consent was raised to 18 years from 16 years), sexual and gender-based violence (SGBV), early and unintended pregnancy, and provision of age appropriate comprehensive SRH information and services. During this period, Kenya also committed to the global initiative, FP2020, a partnership established in 2012 that aimed to address the policy, financing, delivery and socio-cultural barriers to women accessing contraceptive information, services and supplies [28].

Apparent in the late-MDG period was the amendment of the constitution which came as a result of the political dispute stemming from the 2007 national election [29]. The previous constitution which heavily borrowed from the British Common Law saw barriers to many AYSRH reform policies in the pre- and early-MDG period [30]. Although the development of the new constitution started in 1990, the 2007/08 post-election violence and increased international pressure for reforms catalysed its inception. For the first time, the revised 2010 constitution recognised RH as a fundamental human right. It removed barriers that hindered access to health services including RH, set the age of consent to marry at 18 years and protected children (17 years and under) and youth (15–35 years) from abuse, neglect, harmful cultural practices and all forms of violence.

Although the SRH needs of adolescents and young people were not explicitly mentioned in the 2010 revised Constitution except only broadly in terms of rights, it paved way for various policies that specifically addressed the SRH nuances that were within the confines of the 2010 Constitution. The Constitution established the right to RH care within the law, and this saw the development of the first ever government initiative of the Reproductive Health (RH) Communication Strategy in 2010 that focused not only on FP as did most RH initiatives in the previous MDG periods, but included AYSRH as well, with oversight and supervision of implementing partners from the Department of Reproductive Health (DRH) of the Ministry of Health. Unlike the previous MDG periods, this was the first time that the government spearheaded an AYSRH initiative. Noteworthy was the government’s involvement in the national campaign’s RH/FP and Social and Behaviour Change Communication (SBCC) strategy that included IEC materials for including an online component for delivery of AYSRH information [31]. The ministries of Health, Education, Communication and Youth Affairs all worked collaboratively for the first time supporting use of ICT in delivery of AYSRH information, including the implementation of the Education re-entry policy and supporting of school health programs, strengthening partnerships to provide SRH information and services in schools unlike the previous periods where there was no coordination amongst the ministries on AYSRH. This late MDG period also saw the integration of HIV/AIDS with SRH issues for adolescents and young people that was not done in the previous periods, focusing on human rights-based communication in order to ensure provision of adequate information and universal access to RH services for adolescents and young people. The RH strategy of 2010 was also the first ever to include the marginalised adolescents such as those out-of-school, the very young (10–14 years), married adolescents and adolescents living in the streets.

Majority of the policy documents in the late MDG period were informed by the amended Constitution of Kenya and the Vision 2030 strategy. The new Constitution in 2010 also relaxed some of the restrictions on abortion in Kenya, which saw the revision of the National family planning guidelines for service providers in 2010 and guidelines for provision of safe abortion in 2012, so as to make provisions available for safe abortion [7]. While services for AYSRH in the pre- and early-MDG period were non-existent in government facilities, the late MDG period, through the RH rights initiative provided SRH services to adolescents and young people through public and private sector, non-governmental organisations (NGOs), community-based organizations (CBOs) and Faith based organizations (FBOs). The collaboration between NGOs, CBOs and the government were particularly pronounced in nationwide programs- Families Matter! Programme and Youth for Youth (Y4Y) program targeting both very young adolescents and the youth (9-24 years) in promoting ASRH and rights and strengthening the education sector response on sexual risk reduction [32, 33].

Nonetheless, RH as a human right in the constitution opened space for NGOs to fill the void in AYSRH provision of services which collaborated with relevant government line ministries in the implementation of AYSRH programmes. The Ministry of Youth Affairs and Sports (MOYAS) in particular, was the first ministry established in the early MDG period that specifically had a focus on youth, mainly to support the achievement of the MDGs by addressing socio-economic challenges faced by youth in the country [34]. While the pre- and early-MDG periods saw NGOs constrained by the policy environment for the provision of AYSRH due to the strong religious and cultural narratives that were in play, the revised constitution broke the policy barriers and saw more NGOs engage in AYSRH in the country and increased collaboration between the government and NGOs as initiatives that employed a multi-sectoral approach were scaled nationwide showing promising signs of sustainability through the involvement of relevant ministries [35].

AYSRH programmes have since been aligned with the country’s Vision 2030 goals, the revised 2010 constitution and the RH communication strategy that facilitated behaviour change of individuals at family/household level including sensitization of AYSRH through IEC materials, interactive community theatres, parent-youth interactions, road shows, community video centres, community based youth friendly centres, use of television and radio to reach both adolescents and young people and the community. Unlike the previous MDG periods, this later period saw more participatory development communication, advocacy, behaviour change, health education in schools and involvement of various stakeholders, including adolescents, parents and guardians of younger adolescents, policy and opinion leaders in the education system, health service providers, community leaders, religious groups, opinion leaders and social networks.

While the 2003 ARH&D Policy’s focus was primarily on development, the 2015 Policy revision brought ASRH and rights issues into the country’s mainstream health and development agenda (in line with the country’s national goals- Vision 2030 and 2010 constitution), so as to enable a more directed focus on young people’s sexual and reproductive health needs. For the first time in 2015, the word ‘sexual’ was included in the title of the adolescent specific reproductive health policy and included adolescents in the development process which was not the case in the previous policy. The changing international, regional and national legislative and policy environment with regard to adolescents also facilitated the revision of the policy that sought to address continuing and emerging issues such as advances in information, communication and technology (ICT) and the resultant exposure to materials and practices that influence young people’s behaviour; including high incidence of poverty, increased HIV incidence and prevalence and harmful traditional practices that negatively impact adolescent health.

## Discussion

The assessment in this paper has shown that AYSRH policies and programmes evolved progressively during the pre- and within the MDG period in Kenya. Both the pre- and MDG periods were characterized by issue-based national policies and programmes, amidst strong political, cultural and religious barriers to realization of AYSRH. The pre-MDG period was dominated by national policies and programmes targeted at reducing the high population growth and fertility rates, while the early to mid-MDG period was characterized by HIV/AIDS related national development priorities. Strong political will and commitment to advance the SRH rights of adolescent girls and young women in the country became more apparent towards the latter part of the MDG period.

During the pre- and early to mid MDG period, the narratives surrounding issues of AYSRH were underpinned on moral, cultural and religious grounds, promoted particularly by religious leaders that they should abstain from pre-marital sexual activity. Together with the deep-rooted socio-cultural beliefs and the subjugation of women in the pre-MDG period, the policies did not really achieve what they were intended to. The unsupportive political context and dominant moral and cultural narratives dominated adolescents’ and young people’s SRH debates, blocking any reforms that would cater to their SRH needs. Thus, despite civil society organisations advocating for AYSRH throughout the pre-MDG period, no policy reforms were realised to address AYSRH issues in the country within that period. The struggle between the global narratives advocating for SRH rights and the country’s socio-cultural and political context at the time was eminent.

The early part of the MDGs was dominated by HIV specific national policies and programmes due to the high HIV incidence and prevalence during the early 2000s. Similar to the pre-MDG period, the early MDG period saw strong religious opposition to AYSRH. Religious and community leaders relaxed their stance on sexuality education and actively participated in drafting of some policy instruments. However, the moral and cultural narrative remained, with most of the sexual prevention strategies for adolescents and young people being focused on abstinence. Medical narratives dominated organizations that attempted to advocate for AYSRH throughout the early MDG period, taking advantage of the fight against HIV in the country.

A more progressive political and social-cultural space within the early-MDG period was also due to a change in government administration, which saw even more progressive policies developed that would address AYSRH including the first adolescent reproductive and development policy of 2003, strategies on sexual violence and return to school policy for adolescent mothers, among others. However, this period was also characterized by sustained hegemonic masculinity, especially where men’s interests were privileged while marginalizing women’s needs as was seen in the development of the Sexual Offences Act. This was quite evident as well in the male dominated Senate and National Assembly. Various scholars have argued that there is a correlation between women’s participation in parliament and representation of issues affecting women in parliament, where policies concerning women are more likely to be passed if there are more women, than in a male dominated parliament [36, 37].

The late MDG period saw the refocus on SRH issues due to a stall in fertility and sustained early childbearing in the country. The efforts toward the end of this period began to address SRH issues as well, which were further advanced at the end of the MDG period. The 2003 ARH&D policy was revised in 2015. In both the early to the late MDG period, success was driven by the level of political commitment and will, which took into account the social, economic and cultural context and made the necessary resources available.

## Conclusion

The national policies were being formulated within the complexity of competing goals at the national and international arena as was seen in the pre- MDG period. Crichton (2008) argued that contextual factors such as political and international influences and the degree of controversy (including inclusion of stakeholders) interact to determine successes or failure of a policy [2]. As has been seen throughout Kenya’s history up until the revised 2015 AYSRH policy, strong cultural and religious narratives were instrumental in blocking policy reforms for AYSRH in the pre- and early MDG period. However, most of the AYSRH policies, strategies and programmes were catalysed by the adoption of the MDGs that emphasized development issues. The global goals of the MDGs provided a political impetus and framework for galvanizing the local community to work on adolescents and young people’s issues particularly as it pertained to their SRH. Although the division of the religious network in the late MDG period saw the realization of the RH rights in the constitution, the issue of AYSRH is still a contested topic in the country.

The findings reveal that political will does not necessarily translate into domestic acceptance of AYSRH services to adolescents or resource mobilization, which is critical for the sustainability of AYSRH programmes and to keep the positive momentum in improving AYSRH. The results highlight how dominant narratives of social morals and religious purity influence the translation of global goals and commitments to practice at country level. It demonstrates that an understanding of these dominant narratives is important in facilitating and promoting a conducive environment for policy debate and development of SRH programmes for adolescent girls and young women. The global agenda and national policy and strategy development for SRH of adolescent girls and young women should be cognizant of the dominant discourses and narratives that impact on them during the life course, as well as, the role of the different actors in the space.

In summary, and while not exhaustive, this paper has identified several factors pertinent in the translation of global commitments on AYSRH such as SDGs, into national action and practice. These include:

i. Recognition of reproductive health as a human right, and in line with WHO’s advancement of health for all in all its forms (SDG 3).
ii. Country context is important, and global commitments need to take this into account while translating and, in assessing country performance in adaptation and adoption of policies.
iii. Sustained political commitment and will, taking into account the social, economic and cultural context is important. Thus, engagement of all key partners – policy makers, community and religious leaders, donors, civil society and young people themselves - is imperative for success.
iv. Allocation of necessary resources (finances, staff and infrastructure) as evidence of increased national commitment and involvement on the issue.
v. An institutional framework to facilitate coordination, monitoring and accountability is necessary.

While this paper contributes to an understanding of the SRH environment of adolescent girls and young women in Kenya against the back drop of MDGs, it is also very timely as it comes in less than 5 years of the adoption of the 2030 SDG agenda, which has a strong emphasis on adolescents’ and young women’s empowerment, as well as, the adoption of various global agenda on advocacy, such as *Together for Girls, Rise Up, Gender and Adolescents: Global Evidence (GAGE*, *The Global Strategy on Women’s, Children’s and Adolescent Health, 2016–2030 and, Family Planning 2020* -a global partnership to empower women and girls by investing in rights-based family planning, all of which focus on girls and women’s SRH issues, gender equality and empowerment, including sexual violence. These initiatives at the global level have the capacity to translate the broad endorsements into specific policy, programme, and financial commitments; and to act on them [38]. The 2019 ICPD plus 25 Summit in Nairobi reiterated these sentiments with over 1000 commitments made by various stakeholders on programme and policy action, including financial resources [39].

The evolution of all of these advocacy initiatives in the recent past underscores the need for recent evidence and updates to inform the ongoing agenda on adolescents’ and women’s, health and their well-being. Given the emphasis on rights-based approaches, one should be cognizant in employing gender transformative approaches to also reach vulnerable groups of adolescents where gender inequality creates additional barriers to comprehensive access to SRH information and services at the micro and macro level. This paper, therefore, contributes to critical evidence needed for translating these global advocacy commitments to practice at national and sub-national levels in Kenya and beyond, including in other developing countries that share similar characteristics.

### Limitations

This assessment relied on published, administrative and grey literature. Perspectives from key informant interviews would have been useful to further elaborate on some of the findings; However, this was not possible due to time and financial constraints. Given this, we aim to conduct a follow up study that will disentangle the nuances stemming from this assessment with a qualitative angle.

The assessment focused on national level policies in Kenya and thus limits the extent to which the results can be generalized to the decentralized level. Moreover, the assessment of the evolution of AYSRH reforms in Kenya did not look at more in-depth implementation of the AYSRH policies and programmes at the grassroots level, thus unable to know the dynamics of these policies and programmes at those levels.

Despite these limitations, the assessment and analyses in three phases (pre-, early to mid and late-MDG periods) provide pertinent knowledge in the evolution of the policy and programme environment for AYSRH in Kenya and important for informing the translation of the 2030 SDG agenda in the country given the social and moral narratives surrounding AYSRH issues. It also provides important lessons for other countries with a similar context to Kenya when trying to implement global goals against competing domestic priorities and socio-cultural constraints to AYSRH.

## Data Availability

Not Applicable.

## Abbreviations

AYFS: Adolescent and Youth Friendly Services
ARH&D: Adolescent Reproductive Health and Development
AYSRH: Adolescent and Youth Sexual and Reproductive Health
CBO: Community- Based Organizations
CHAK: Christian Health Association of Kenya
CSA: Centre for the Study of Adolescents
CSE: Comprehensive Sexuality Education
CSO: Civil Society Organisation
DRH: Department of Reproductive Health
EFA: Education for All
FBO: Faith Based Organizations
FGM: Female Genital Mutilation
FP: Family Planning
ICPD: International Conference on Population and Development
ICT: Information, Communication and Technology
IEC: Information, Education and Communication
KARP: Kenya Adolescent Reproductive Health Programme
MDG: Millennium Development Goals (MDGs)
MOYAS: Ministry of Youth Affairs and Sports
MP: Member of Parliament
NACC: National Aids Control Council
NASCOP: National Aids and STI Control Program
NCPD: National Council for Population Development
NGO: Non-government Organisation
PEPFAR: President’s Emergency Plan for AIDS Relief
POA: Plan of Action
RH: Reproductive Health
SBCC: Social and Behaviour Change Communication
SDG: Sustainable Development Goal
SGBV: Sexual and Gender-Based Violence
SRH: Sexual and Reproductive Health
UN: United Nations
YFS: Youth Friendly Service

## References

[1] Shiffman J, Smith S (2007). Generation of political priority for global health initiatives: a framework and case study of maternal mortality. Lancet.

[2] Crichton J (2008). Changing fortunes: analysis of fluctuating policy space for family planning in Kenya. Health Policy Plan.

[3] Chimbwete C, Cotts S, Zulu E (2005). The evolution of population policies in Kenya and Malawi. Popul Res Policy Rev.

[4] Ajayi A, Kekovole J, Jain A (1998). Kenya’s population policy: from apathy to effectiveness. Do population policies matter? Fertility and politics in Egypt, India, Kenya and Mexico.

[5] Heisel DF, Robinson WC, Ross JA (2007). Family Planning in Kenya in the 1960s and 1970s. The Global Family Planning Revolution: Three Decades of Population Policies and Programs.

[6] Centre for the Study of Adolescence [CSA] (1995). Adolescence in Kenya: The Facts. Nairobi: The Centre for the Study of Adolescence.

[7] Oronje RN (2013). The Kenyan national response to internationally agreed sexually and reproductive health and rights goals: a case study of three policies. Reprod Health Matters.

[8] United Nations Population Fund [UNFPA] (2004). Programme of action adopted at the international conference on population and development, Cairo 5-13 September 1994.

[9] United Nations Convention on the Rights of the Child [UNCRC]. Convention on the rights of the child. 1989. https://downloads.unicef.org.uk/wp-content/uploads/2016/08/unicef-convention-rights-child-uncrc.pdf?_ga=2.127521742.277387518.1583406142-1259528725.1583406142. Accessed 25 Feb 2020.

[10] United Nations. Beijing platform for action. 1995. https://www.un.org/en/events/pastevents/pdfs/Beijing_Declaration_and_Platform_for_Action.pdf. Accessed 25 Feb 2020.

[11] United Nations [UN] (2003). The Convention on the Elimination of All Forms of Discrimination against Women (CEDAW) and its Optional Protocol.

[12] Njau W (1991). Traditional sex education in Africa: The case of Kenya. Presented at the 1st Inter-African Conference on Adolescent Health in Africa.

[13] Wanyama GE, Simatwa ME (2011). Prospects and challenges in the implementation of re-entry policy of girls in secondary schools in Kenya: a case study of Emuhaya District. Educ Res.

[14] Evelia H, Wanjiru M, Obare F, Birungi H (2011). Ten years of the Kenya adolescent reproductive health project: what has happened? APHIA II OR project in Kenya.

[15] Oronje NR, Undie C, Zulu E, Crichton J (2011). Engaging media in communicating research on sexual and reproductive health and rights in sub- Saharan Africa: Experiences and lessons learned. Health Res Policy Syst.

[16] Mpesha N (2000). Curbing Dropout: Re-entry Programme for teenage Mothers. The Case of Kenya.

[17] Lloyd CB, Mensch BS (2008). Marriage and childbirth as factors in dropping out from school: an analysis of DHS data from sub-Saharan Africa. Popul Stud.

[18] Centre for the Study of Adolescence [CSA] (2008). Down the Drain: Counting the Costs of Teenage Pregnancy and School Drop out in Kenya.

[19] Macharia JW, Kessio KD (2015). Investigation of re-entry of student mothers in secondary schools in Kenya. Int J Human Soc Sci Educ.

[20] Trust. Kenyans clash in debate to bring sex education into schools. 2011. http://news.trust.org//item/20111021105200-su3dl/. Accessed 4 Jan 2019.

[21] Ogot BA (2004). Politics and the AIDS epidemic in Kenya 1983–2003.

[22] Ndungu N (2008). Legislation for sexual violence in Africa: Preparing and delivering evidentiary requirements.

[23] Hansard (2006). National Assembly Official Report - April 26, April 27, May 2, and May 31.

[24] Ngethe NP (2014). Family planning in Kenya: A review of National and District Policies and Budgets.

[25] Onyango-Ouma W, Ndung’u N, Baraza N, Birungi H (2009). The making of the 2006 Kenya sexual offenses act: behind the scenes.

[26] Waituru M (2013). Lessons from the Implementation of MDGs in Kenya: Options for a Post-2015 Framework. Institute of Development Studies Bulletin.

[27] Crane BB, Dusenberry J (2004). Power and politics in international funding for reproductive health: the US global gag rule. Reprod Health Matters.

[28] Family Planning [FP2020] (2011). London Summit on Family Planning, July 11.

[29] Committee of Experts of Constitutional Review. Review of the Constitution on 8th January 2010 by the Committee of Experts on Constitutional Review pursuant to Section 32 (1) (c) of the Constitution of Kenya Review Act, 2008. 2010. https://katibaculturalrights.files.wordpress.com/2016/04/final_report_on_the_reviewd_draft1.pdf. Accessed 25 Feb 2020.

[30] Robila M (2014). Handbook of family policies across the globe.

[31] Republic of Kenya (2010). Reproductive health communication strategy implementation guide 2010–2012.

[32] Center for Discease Control and Prevention [CDC]. The Families Matter! Program. 2014. https://www.cdc.gov/globalaids/publications/fmp-2-pager-final-jan-2014.pdf. Accessed 25 Feb 2020.

[33] Tavrow P. Report on youth for youth program in Bungoma District, Kenya. 2008. http://www.csakenya.org/pdfs/y4y_brief.pdf. Accessed 25 Feb 2020.

[34] Ministry of Youth Affairs and Sports [MOYAS]. 2005. http://www.psyg.go.ke/2016-02-05-06-32-50/public-service-youth-affairs/directorate-of-youth-affairs/about-us. Accessed 2 Apr 2019.

[35] Department of Reproductive Health [DRH] (2013). Adolescent and youth sexual and reproductive health evidence-based interventions in Kenya.

[36] Tremblay M (1998). Do female MPs substantively represent women? A study of legislative behaviour in Canada’s 35th parliament. Can J Polit Sci.

[37] Devlin C, Elgie R (2008). The effect of increased women’s representation in parliament. The case of Rwanda. Parliam Aff.

[38] Chandra-Mouli V, Plesons M, Sullivan E, Gonsalves L, Say L (2018). 38.8 million additional modern contraceptive users: this, in fact, is “a never-before opportunity to strengthen investment and action on adolescent contraception.”. Reprod Health.

[39] International Conference for Population Development [ICPD 25], Nairobi Summit. [cited 2019 Dec 20]. Available from: https://www.nairobisummiticpd.org/commitments. Accessed 20 Dec 2019.

